# The mediation role of physical fitness in association between muscle-strengthening physical activities and its component with blood pressure among young adults: considering gender and abnormal blood pressure as moderators, moderate-vigorous physical activity, sleep behavior, sedentary behavior, mental wellbeing and BMI as covariates

**DOI:** 10.3389/fcvm.2023.1158893

**Published:** 2023-09-20

**Authors:** Mumtaz Maulana Hidayat, Denny Agustiningsih, Rahmaningsih Mara Sabirin, Rakhmat Ari Wibowo

**Affiliations:** Department of Physiology, Faculty of Medicine, Public Health and Nursing, Gadjah Mada University, Yogyakarta, Indonesia

**Keywords:** mediation analysis, physical fitness, muscle strengthening, physical activity, blood pressure, young adults

## Abstract

**Background:**

Global burden of hypertension among young people continues to increase. There have been many studies examining the effect of aerobic and muscle-strengthening physical activity on blood pressure, many of them didn't consider interdependence between them. Conflicting results of health-related fitness, particularly handgrip strength, as intermediate outcomes of muscle-strengthening physical activity on blood pressure also emerged. This research will carry out a mediation-moderation analysis to find out the relationship between muscle strengthening physical activity and blood pressure among young adults by considering health-related fitness and 24-hour movement behavior.

**Methods:**

A cross-sectional study among 221 Indonesian young adults attending a physical activity intervention collected participant's muscle-strengthening physical activity, and 24 h movement behavior, including aerobic physical activity, sedentary and sleep behavior, and mental well-being using validated questionnaires. Mediation and moderation analyses were conducted using Process Macro model 10 on SPSS 25 to investigate the association of muscle-strengthening physical activity on blood pressure, with gender and blood pressure as moderator, mediators consist of handgrip strength, muscle mass percentage and cardiorespiratory fitness. A subgroup analysis was conducted based on participant's cardiorespiratory fitness level.

**Results:**

Volume of muscle-strengthening physical activities in a week have a direct association with systolic blood pressure among prehypertensive male with an effect of 0,00989359 (95% CI 0,0046488 to 0,00336478). Considering its volume as mediator, the frequency of muscle-strengthening physical activity contributed to a significant direct effect on diastolic blood pressure in both genders, but the duration of MSPA has a significant direct effect on systolic blood pressure in male subjects. There is no component of physical fitness that provides a significant mediating effect. After a subgroup analysis, the relationship between MSPA Volume and blood pressure is not significant for individuals with a high level of cardiorespiratory fitness.

**Conclusions:**

This study shows that increased participation in muscle strengthening physical activity, especially in subject with low cardiorespiratory fitness, could increase blood pressure in prehypertensive young adult male population without mediation by physical fitness. Further research is needed to investigate other mechanisms that influence this relationship.

## 1. Introduction

The prevalence of hypertension, a non-communicable condition, is quite high and is constantly rising. From 1990 to 2019, the number of persons with hypertension more than doubled, from 600 million to more than 1 billion (1). In just a few years, the prevalence of hypertension among Indonesian adults between the ages of 18 and 24 has nearly doubled (2). Given that hypertension, especially in young people, can continue to rise and constitute a major financial burden on the world's healthcare system. The average total costs of hypertension for all the studied countries, calculated per person, amounted to 630.14 USD, with direct costs of 1,497.36 USD and indirect costs of 282.34 USD (3). That should be a serious worry.

The high rate of hypertension at a young age is inseparable from the reluctance of young people to do physical activity (4, 5). Various studies have shown the benefits of physical activity in maintaining health and controlling non-communicable diseases such as hypertension. Aerobic physical activity and muscle strengthening physical activity are the most commonly associated with blood pressure. Muscle-strengthening physical activities (MSPA) deserve more attention because health surveys in various countries also show that only 10%–30% of adults do MSPA according to recommendations (6). MSPA are also rarely the focus of promoting physical activity for public health in various countries (7).

Although there have been many studies looking for a relationship between MSPA and blood pressure, many of them didn't consider the interdependence between them. While health- related fitness components such as muscle strength can be intermediates between MSE and blood pressure, some studies show inconsistent results. MSPA is generally associated with a lower risk of high blood pressure, but several studies have shown that higher blood pressure is associated with higher grip strength through mechanisms that are unclear. Therefore, it is necessary to do an analysis to assess the relationship between various factors (8, 9, 10).

In addition, various health-related factors that can affect blood pressure conditions, such as age, mental health, sleeping behavior, and sedentary behavior are also rarely considered. Age is globally known as one of the biggest risk factor in high blood pressure incidence, including in Indonesia (11). Mental health condition, such as anxiety or depression are also associated with the incidence of high blood pressure in young adults by unclear mechanism (12, 13). There is strong evidence of an association between sleep disturbance and increased risk of blood pressure, especially in young population (14, 15). Sedentary behavior in adult population is also associated with elevated blood pressure (16). The relationship between blood pressure and all the above factors encourages us to control them as covariates in this study.

Moreover, gender and abnormal blood pressure as potential moderators. There are several studies that show differences in response to physical exercise between men and women. Systolic Blood Pressure (SBP) and Diastolic Blood Pressure (DBP) reduction with muscle strengthening exercises differs statistically between men and women (17). Another study showed statistically significantly more pronounced DBP and mean arterial pressure (MAP) reductions from isometric resistance training among males than females (18). A meta-analysis also showed that the reduction in MAP due to muscle strengthening exercises was greater in subjects with hypertension than in subjects with normal blood pressure (19).

Given that there is still a knowledge gap where there are not many mediation analysis studies that link MSPA and blood pressure with consideration of the interdependence connection between them, this research will carry out a mediation-moderation analysis to find out the relationship between muscle strengthening physical activity and blood pressure by considering gender and abnormal blood pressure as moderator, muscle strength, muscle mass percentage and cardiorespiratory fitness as mediator and moderate-vigorous physical activity, age, sleep behavior, sedentary behaviour, mental wellbeing and BMI as covariates.

## 2. Material and methods

### 2.1. Study design and setting

We conducted a cross-sectional study taking place at Gadjah Mada University, Yogyakarta, Indonesia. This study was conducted to see the mediating role of physical fitness in the relationship between MSPA and its domain with blood pressure in the young adult population by considering moderate-vigorous physical activity, age, mental health, sleep behavior, and sedentary behavior. Data collection was carried out for 1 month in November-December 2022. The study protocol was approved by the medical and health research ethics committee of the Faculty of Medicine, Public Health, and Nursing at UGM (approval number: KE/FK/1382/EC/2022). This study is a sub-study of a quasi-experimental study entitled “*Effectiveness of a virtual challenge with a social component in promoting physical activity, hydration, and fruit consumption among university students*.” This study protocol has been registered with the Thai Clinical Registry with the registry number TCTR20230106003. We reported our current study following the strengthening the reporting of observational studies in epidemiology (STROBE) statement and A Guideline for Reporting Mediation Analyses of Randomized Trials and Observational (20, 21).

### 2.2. Participants and sample size

All Gadjah Mada University students, including those pursuing vocational, undergraduate, master, and doctoral degrees, were qualified to take part in the study. Subject age was limited to the range of 18–25. The study excluded participants who had experienced a heart attack within the previous three months, were recovering from surgery, and had physical or mental impairments that precluded them from taking part in all tests. Convenience sampling technique was adopted in this investigation. G*Power analysis was used to calculate the sample size to examine the link between the volume of muscle-strengthening activities and blood pressure (22). Using a power of 0.8, an alpha level of 0.05, and an effect size of 0.2, the minimal number of samples is 153 (23). Out of a total of 412 eligible prospective subjects, 155 did not give their consent to participate. 257 subjects were checked for eligibility, and 22 were excluded for not attending fitness checks. Six subjects had musculoskeletal problems that were contraindications for fitness checks. 231 subjects were confirmed as eligible for the study. After there were 10 missing and invalid data, 221 subjects were included in the analysis. The study flow diagram follows previous study.

### 2.3. Effect of interest

The primary aim of this study was examining the direct effect of volume of MSPA on blood pressure. In addition, the study also aimed to analyze the indirect effect of various physical fitness components as potential mediators in the relationship between them by considering the role of sex and abnormal blood pressure as moderators.

This study not only wants to see the direct effect of volume of MSPA on blood pressure but also the indirect effect of various physical fitness components as potential mediators in the relationship between them. This study also wants to see the role of sex and abnormal blood pressure as moderators.

### 2.4. Variables and causal assumption

**Figure 1:**
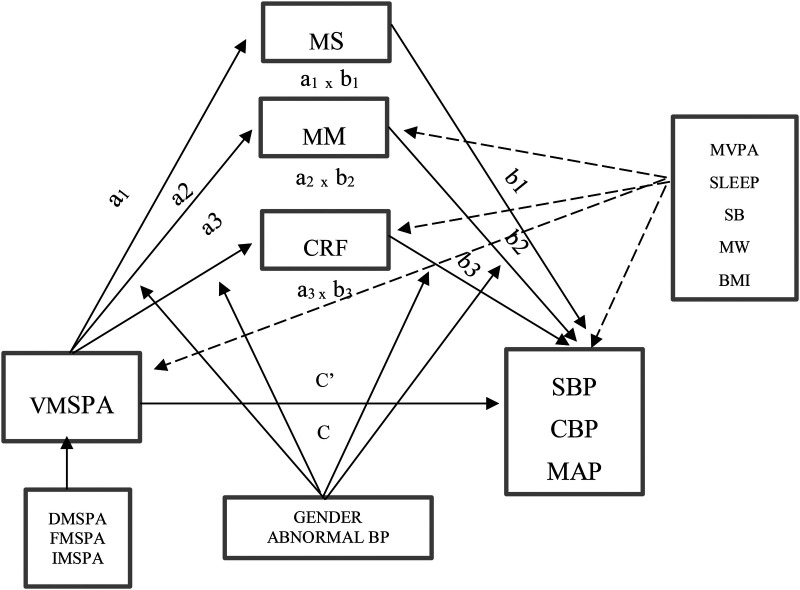
Causal model representing moderated-mediation analysis of association between muscle-strengthening physical activity with blood pressure considering covariates. a, effect of independent variable on mediator; b, effect of mediator on outcome; c’, direct effect of independent variable on outcome; c, total effect of independent variable on outcome; VMSPA, Volume of Muscle-Strengthening Physical Activity; DMSPA, Duration of Muscle-Strengthening Physical Activity; FMSPA, Frequency of Muscle-Strengthening Physical Activity; IMSPA, Intensity of Muscle-Strengthening Physical Activity; MVPA, Moderate-Vigorous Aerobic Physical Activity; MW, Mental Well-being; MS, Muscle Strength; MM, Muscle Mass Percentage; CRF, Cardiorespiratory Fitness; BMI, Body Mass Index; SLEEP, Sleep Quality and Quantity; SB, Sedentary Behavior; SBP, Systolic Blood Pressure; DBP, Diastolic Blood Pressure; MAP, Mean Arterial Pressure. - -, covariates; __, moderators.

We used a modeling framework to examine whether physical fitness mediates the relationship between MSPA volume and blood pressure (Figure 1). MSPA served as an independent variable, while systolic blood pressure, diastolic blood pressure, and mean arterial pressure served as dependent variables. We also included sedentary behavior, moderate-to-vigorous aerobic physical activity, and sleep behavior as covariates. The model is created with the assumption that independent variables have a significant correlation with the outcome, independent variables have a significant correlation with the mediator, and mediator has a significant correlation with the dependent variable. We investigated the total effect (path c) and direct effect (path c') in the figure above using linear regression with the Hayes model. Path a showed the correlation between the independent variables (MSPA) and the mediators (muscle mass, muscle strength, and CRF). Path b represents the correlation between the mediators (muscle mass, muscle strength, and CRF) and the dependent variable (BP). After adjustment for confounders, the a-b pathway represents the indirect effect of MSPA, which is mediated by muscle mass, muscle strength, and CRF. The mediated effect is a multiplication of effects a and b. Therefore, the direct effect c' represents the independent variable's effect after considering the mediated effect.

### 2.5. Outcome measure

#### 2.5.1. Main outcome: blood pressure

After a minimum of five minutes of rest, SBP and DBP were assessed in a sitting position using a certified digital instrument (Omron HEM 705CPINT, USA) (24). We measure twice, one minute apart, and then average the two results. Blood pressure measurements were obtained from 8 to 10 a.m. to minimize circadian effects.

#### 2.5.2. Main predictor: volume of muscle strength physical activity

The volume of Muscle Strength Physical Activity (MSPA) for a week was measured by the Muscle Strengthening Exercise Questionnaire (MSEQ), which consists of questions about the duration, intensity, frequency, and domain of muscle strengthening exercises (24). MSPA is expressed in MET-minutes per week.

#### 2.5.3. Potential mediator

##### 2.5.3.1. Muscle strength

Muscle strength was assessed with a Camry handgrip dynamometer (Model EH101). All subjects were asked to sit down while holding the dynamometer in the dominant hand with their arms at a 90degree angle with the body without squeezing the arms against the body. Participants are allowed to do one trial. As in the previous study (10, 25), three trials were performed, resting 60 s between measurements on the same hand. The dynamometer was adjusted to the size of the participant's hand before measuring grip strength by turning the adjusting knob right or left to get an ideal grasp. All three scores are averaged for analysis. Grip strength is expressed in kilograms.

##### 2.5.3.2. Muscle mass

Omron Karada Scan HBF 375 Body Fat Composition Monitor Used to measure body weight in kilograms (kg) and percentage of skeletal muscle mass.

##### 2.5.3.3. Cardiorespiratory fitness

Cardiorespiratory fitness examined by the 6-Minute Walking Test (6MWT) method. The result of the 6MWT distance in meters is converted to an estimate of V˙O2max by the formula (26). V˙O2max is expressed in numbers, with units of ml/kg/minute. The Vo2max level is categorized as good if it is above 40 for women and above 50 for men. In the sensitivity analysis, we consider the VO2max category. All physical fitness examinations are carried out in the morning (08.00–12.00 am) to avoid the effects of circadian rhythms.

#### 2.5.4. Covariate and other variables

Covariates consist of Moderate-Vigorous physical activity (MVPA), sedentary behavior, sleep behavior, mental wellbeing, and BMI. MVPA, sedentary behavior, and sleeping habits are measured from questions on a questionnaire that has been tested for reliability and validity in our unpublished study (27). MVPA is expressed in Met-Minutes per week. While sleep behavior is expressed in Score (28, 29) and sedentary behavior expressed in minutes per week. Mental wellbeing was measured using the shortened Warwick-Edinburgh Mental Well-being Scale (WEMWBS) which has been translated and validated in Bahasa Indonesia (28, 29). BMI is obtained from body weight in kg divided by height in m2 and expressed in number.

### 2.6. Statistical analysis

Data were displayed using descriptive statistics in the form of a mean and standard deviation. The distribution of categorical variables is presented using frequency and proportion. If data is normally distributed, then Pearson correlation was used to see the relationship between volume of muscle strengthening exercises, blood pressure, muscle strength, and percentage of muscle mass. If the data is not normally distributed, then an alternative is using Spearman's correlation. The percentile bootstrapping linear regression using Hayes model was used to assess the mediating effect of muscle strength and muscle mass percentage. We present the results of the mediation-moderation analysis with data that has been adjusted by the covariates and without adjustment. A sensitivity analysis was carried out by considering the level of cardiopulmonary fitness. All analyses were performed using the SPSS version 25 application with a significance level of 0.05.

## 3. Result

257 undergraduate students who were among the 412 participants in the virtual challenge decided to take part in the study and completed the questionnaire. Ninety percent of them (235) attended the health and fitness examination, but 4 of them had an absolute contraindication to fitness measurement, resulting in a total of 231 study subjects. After excluding 5 missing and 5 invalid data, a total of 221 subjects were finally included in the analysis. Based on Table 1, the mean age was 19.25 years old, the majority of subjects were 19 years old (121, or 54.8%), and the rest were in the age ranges of 18 years and 20–23 years. The majority of subjects were female, namely 143 people (64.7%) and the remaining 78 men (35.3%). The data in Table 1 show that the participants' average volume of muscle-strengthening physical activity per week is still below moderate-vigorous aerobic physical activity, where the ratio is three times. As many as 131 subjects (59.3%) had systolic and diastolic blood pressure within the normal range, while the remaining 90 people (40.7%) were in the high blood pressure category (systolic >120 and/or diastolic >80 mmHg).

**Table 1:** Participant characteristics.

	Minimum	Maximum	Mean	Std. Deviation
Age (years)	18	23	19.3	1.0
Weight (kg)	36.9	108.0	61.5	13.4
Height (centimeter)	145.0	182.0	161.7	8.2
BMI (kg/m^2^)	15.9	41.2	23.4	4.3
Muscle Mass Percentage (%)	7.6	40.2	28.5	4.9
Body Fat Percentage (%)	5.5	43.1	26.9	7.6
Muscle Strength (Kg)	0.4	2.5	1.1	0.4
Cardiorespiratory Fitness (ml/kg/min)	15.4	71.5	36.4	8.5
Volume of MSPA (VMSPA) (Met-Minutes)	0	8,960.0	488.3	1,274.5
Volume of MVPA (Met-Minutes)	0	34,160	1,357	2,994.6
Sedentary Time (Avgsb) (min)	10	1,142.9	338.2	243.4
Sleeping Behaviour (PSQI) [Score (0–21)]	2	11	5.1	1.6
Systolic Blood Pressure (SBP) (mmHg)	84	150	114	13.7
Diastolic Blood Pressure (DBP) (SBP) (mmHg)	56.5	102.5	75.1	8.1
Mean Arterial Pressure (MAP) (SBP) (mmHg)	69	117	88.1	8.9

**Figure 2:**
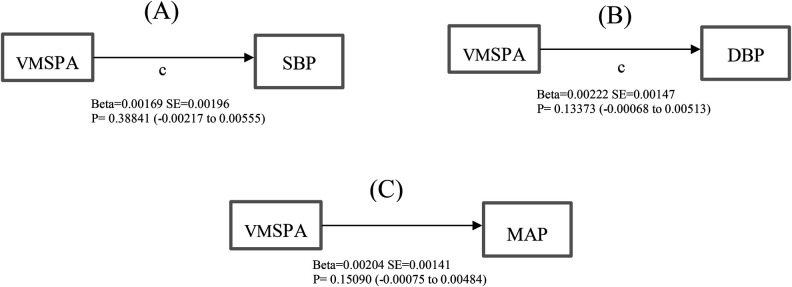
Total effect (path c) of muscle-strengthening physical activity volume on (**A**) systolic blood pressure (**B**) diastolic blood pressure (**C**) mean arterial pressure.

According to the R2 of the unadjusted models (Figure 2), muscle-strengthening physical activity explains the variation in blood pressure readings in the population by 66.27% for systolic blood pressure, 48.72% for diastolic blood pressure, and 60.75% for MAP, MSPA, on the other hand, accounted for 69.16%, 49.79%, and 62.36% of the variation in the population's systolic, diastolic, and MAP blood pressure readings in the table. Therefore, tables were used in explaining the mediation-moderation analysis.

Looking at the results of the adjusted model that included covariates (Tables 2–5), it is clear that muscle-strengthening physical activity has a significant direct effect on systolic blood pressure in the male population with high blood pressure (*β* = 0.00191, 95% CI 0.00046 to 0.00335, *t* = 2.60425, *p* = 0.00987), but not in women (*β *= 0.00015, 95% CI −0.00339 to 0.00370, *t* = 0.08623, *p* = 0.93136). Looking at these data, it can be estimated that every 625 min of 4 MET (moderate intensity) muscle-strengthening physical activity in 1 week is associated with an increase in systolic blood pressure of 5 mmHg in the male population that has high blood pressure. Neither muscle mass, muscle strength nor cardiorespiratory fitness has a mediating effect on the relationship between MSPA volume and blood pressure.

**Table 2:** Direct and indirect effect of muscle-strengthening physical activity volume (VMSPA) on BP parameter in male group with Abnormal blood pressure.

VMSPA	BP Parameter	Direct Effect VMSPA → BP	PF at Mediator		Indirect Effect VMSPA → PF → BP
	SBP	Beta: 0.00191 **p: 0.00959**	MS	SBP	Beta: −0.00003
	DBP	Beta: −0.0043 p: 0.43534		DBP	Beta: −0.00000
	MAP	Beta: 0.00034 p: 0.51224		MAP	Beta: −0.00001
			MM	SBP	Beta: −0.00001
				DBP	Beta: 0.00003
				MAP	Beta: 0.00002
			CRF	SBP	Beta: 0.00191
				DBP	Beta: −0.00002
				MAP	Beta: 0.00000

VMSPA, volume of muscle-strengthening physical activity; BP, blood pressure; SBP, systolic blood pressure; DBP, diastolic blood pressure; PF, physical fitness; MAP, mean arterial pressure; MS, muscle strength; MM, muscle mass; CRF, cardiorespiratory fitness.

Bold indicates statistical significant at *p*-value <0.05.

**Table 3:** Direct and indirect effect of muscle-strengthening physical activity volume (VMSPA) on BP parameter in male group with normal blood pressure.

VMSPA	BP Parameter	Direct Effect VMSPA → BP	PF at Mediator		Indirect Effect VMSPA → PF → BP
	SBP	Beta: −0.00005 *p*: 0.92011	MS	SBP	Beta: −0.00000
	DBP	Beta: −0.00000 *p*: 0.98556		DBP	Beta: −0.00000
	MAP	Beta: −0.00002 *p*: 0.95318		MAP	Beta: −0.00000
			MM	SBP	Beta: 0.00000
				DBP	Beta: −0.00001
				MAP	Beta: −0.00000
			CRF	SBP	Beta: −0.00000
				DBP	Beta: −0.00000
				MAP	Beta: −0.00000

VMSPA, volume of muscle-strengthening physical activity; BP, blood pressure; SBP, systolic blood pressure; DBP, diastolic blood pressure; PF, physical fitness; MAP, mean arterial pressure; MS, muscle strength; MM, muscle mass; CRF, cardiorespiratory fitness.

Bold indicates statistical significant at *p*-value <0.05.

**Table 4:** Direct and indirect effect of muscle-strengthening physical activity volume (VMSPA) on BP parameter in female group with Abnormal blood pressure.

VMSPA	BP Parameter	Direct Effect VMSPA → BP	PF at Mediator		Indirect Effect VMSPA → PF → BP
	SBP	Beta: 0.00014 *p*: 0.93604	MS	SBP	Beta: 0.00015
	DBP	Beta: −0.00266 ***p*: 0.05095**		DBP	Beta: 0.00003
	MAP	Beta: −0.00172 *p*: 0.18686		MAP	Beta: 0.00007
			MM	SBP	Beta: 0.00004
				DBP	Beta: −0.00012
				MAP	Beta: −0.00006
			CRF	SBP	Beta: 0.00001
				DBP	Beta: 0.00000
				MAP	Beta: 0.00000

VMSPA, volume of muscle-strengthening physical activity; BP, blood pressure; SBP, systolic blood pressure; DBP, diastolic blood pressure; PF, physical fitness; MAP, mean arterial pressure; MS, muscle strength; MM, muscle mass; CRF, cardiorespiratory fitness.

Bold indicates statistical significant at *p*-value <0.05.

**Table 5:** Direct and indirect effect of muscle-strengthening physical activity volume (VMSPA) on BP parameter in female group with normal blood pressure.

VMSPA	BP Parameter	Direct Effect VMSPA → BP	PF at Mediator		Indirect Effect VMSPA → PF → BP
	SBP	Beta: 0.00182 *p*: 0.93604	MS	SBP	Beta: 0.00011
	DBP	Beta: −0.00224 *p*: 0.07089		DBP	Beta: 0.00003
	MAP	Beta: 0.00000 *p*: 0.07782		MAP	Beta: 0.00005
			MM	SBP	Beta: 0.00005
				DBP	Beta: −0.00017
				MAP	Beta: 0.00005
			CRF	SBP	Beta: 0.00001
				DBP	Beta: 0.00000
				MAP	Beta: 0.07782

VMSPA, volume of muscle-strengthening physical activity, BP, blood pressure, SBP, systolic blood pressure, DBP, diastolic blood pressure, PF, physical fitness, MAP, mean arterial pressure, MS, muscle strength, MM, muscle mass, CRF, cardiorespiratory fitness.

Bold indicates statistical significant at *p*-value <0.05.

Tables 6–8 show the effect and direct effect of MSPA components (which include MSPA duration, frequency, and intensity) on blood pressure outcome via MSPA volume mediation. Of the three components, the frequency of MSPA contributed to a significant direct effect on diastolic blood pressure in both genders (male *p* = 0.02064, 95% CI −1.63213 to −0.13681; female *p* = 0.03221, 95% CI −2.59071 to −0.11597). Meanwhile, the duration of MSPA has a significant direct effect on systolic blood pressure in male subjects (*p* = 0.02087, 95% CI 0.00446 to 0.05389). After looking at the direct and indirect effects in the model above, we conducted a sensitivity analysis by comparing the data on the relationship between VMSPA and blood pressure by grouping subjects based on their level of cardiopulmonary fitness, shown in Tables 9–11.

**Table 6:** Direct and indirect effect of muscle strength physical activity frequency (FMSPA) on BP parameter.

FMSPA		BP Parameter	Direct Effect FMSPA → BP	Indirect Effect FMSPA → VMSPA → BP
	Male (H)	SBP	Beta: 0.047928 *p*: 0.35051	Beta: 0.30126
		DBP	Beta: −0.88447 ***p*: 0.02064**	Beta: 0.10459
		MAP	Beta: −0.42988 *p*: 0.24227	Beta: 0.17015
	Male (n)	SBP	Beta: −1.01443 *p*: 0.19423	Beta: 0.47969
		DBP	Beta: −0.62845 *p*: 0.27711	Beta: 0.16653
		MAP	Beta: −0.75711 *p*: 0.17590	Beta: 0.27091
	Female (H)	SBP	Beta: 0.32201 *p*: 0.70443	Beta: 0.02837
		DBP	Beta: −1.35334 ***p*: 0.03221**	Beta: 0.00985
		MAP	Beta: −0.79489 *p*: 0.19157	Beta: 0.01602
	Female (n)	SBP	Beta: −1.17170 ***p*: 0.04282**	Beta: 0.20680
		DBP	Beta: −1.09732 *p*: **0.01063**	Beta: 0.07179
		MAP	Beta: −1.12212 *p*: **0.00693**	Beta: 0.11679

VMSPA, volume of muscle-strengthening physical activity; BP, blood pressure; SBP, systolic blood pressure; DBP, diastolic blood pressure; PF, physical fitness; MAP, mean arterial pressure; MS, muscle strength; MM, muscle mass; CRF, cardiorespiratory fitness.

Bold indicates statistical significant at *p*-value <0.05.

**Table 7:** Direct and indirect effect of muscle strength physical activity duration (DMSPA) on BP parameter.

DMSPA		BP Parameter	Direct Effect DMSPA → BP	Indirect Effect DMSPA → VMSPA → BP
	Male (H)	SBP	Beta: 0.01253 ***p*: 0.02087**	Beta: −0.01345
		DBP	Beta: 0.00638 *p*: 0.50520	Beta: −0.00830
		MAP	Beta: 0.01389 *p*: 0.12795	Beta: −0.01002
	Male (n)	SBP	Beta: 0.01016 *p*: 0.29516	Beta: −0.01080
		DBP	Beta: 0.00594 *p*: 0.42212	Beta: −0.00667
		MAP	Beta: 0.00735 *p*: 0.29955	Beta: −0.00805
	Female (H)	SBP	Beta: 0.02449 *p*: 0.05439	Beta: −0.01070
		DBP	Beta: −0.00046 *p*: 0.96191	Beta: −0.00661
		MAP	Beta: 0.00785 *p*: 0.39632	Beta: −0.00797
	Female (n)	SBP	Beta: 0.00547 *p*: 0.61023	Beta: −0.00806
		DBP	Beta: −0.00090 *p*: 0.91235	Beta: −0.00497
		MAP	Beta: 0.00122 *p*: 0.87598	Beta: −0.00600

VMSPA, volume of muscle-strengthening physical activity; BP, blood pressure; SBP, systolic blood pressure; DBP, diastolic blood pressure; PF, physical fitness; MAP, mean arterial pressure; MS, muscle strength; MM, muscle mass; CRF, cardiorespiratory fitness.

Bold indicates statistical significant at *p*-value <0.05.

**Table 8:** Direct and indirect effect of intensity of muscle-strengthening physical activity (IMSPA) on BP parameter.

IMSPA		BP Parameter	Direct Effect IMSPA → BP	Indirect Effect IMSPA → VMSPA → BP
	Male (H)	SBP	Beta: −0.59165 *p*: 0.34460	Beta: 0.08991
		DBP	Beta: −0.42158 *p*: 0.36925	Beta: −0.04625
		MAP	Beta: −0.47827 *p*: 0.28907	Beta: −0.00086
	Male (n)	SBP	Beta: −1.04070 *p*: 0.14716	Beta: 0.13832
		DBP	Beta: 0.12560 *p*: 0.81513	Beta: −0.07115
		MAP	Beta: −0.26316 *p*: 0.61009	Beta: −0.00133
	Female (H)	SBP	Beta: −0.00828 *p*: 0.98811	Beta: 0.02345
		DBP	Beta: −0.35025 *p*: 0.40157	Beta: −0.01206
		MAP	Beta: −0.23626 *p*: 0.55560	Beta: −0.00022
	Female (n)	SBP	Beta: −0.45734 *p*: 0.37314	Beta: 0.07186
		DBP	Beta: 0.19692 *p*: 0.60896	Beta: −0.03696
		MAP	Beta: −0.02116 *p*: 0.95433	Beta: −0.00069

VMSPA, volume of muscle-strengthening physical activity; BP, blood pressure; SBP, systolic blood pressure; DBP, diastolic blood pressure; PF, physical fitness; MAP, mean arterial pressure; MS, muscle strength; MM, muscle mass; CRF, cardiorespiratory fitness.

Bold indicates statistical significant at *p*-value <0.05.

**Table 9:** Subgroup analysis with cardiorespiratory fitness classification parameters on the direct relationship between muscle strengthening physical activity volume and systolic blood pressure.

VMSPA → SBP	Sex	R-Sq	Effect	SE	*t*	p	LLCI	ULCI
All	Male	0.6919	0.00191	0.00073	2.60358	**0** **.** **00989**	0.00046	0.00356
	Female		0.00014	0.00180	0.08033	0.93604	−0.00341	0.00369
Good CRF	Male	0.7544	0.0018	0.0011	1.6516	0.1057	−0.0004	0.0040
	Female		−0.0231	0.0340	−0.6791	0.5006	−0.0916	0.0454
Low CRF	Male	0.6539	0.0029	0.0011	2.5316	**0**.**0124**	0.0006	0.0052
	Female		0.0012	0.0023	0.5215	0.6028	−0.0033	0.0057

VMSPA, volume of muscle-strengthening physical activity; SBP, systolic blood pressure; CRF, cardiorespiratory fitness; R-Sq, R-squared; SE, standard error; LLCI, lower limit 95% confidence interval; ULCI, upper limit 95% confidence interval.

Bold indicates statistical significant at *p*-value <0.05.

**Table 10:** Subgroup analysis with cardiorespiratory fitness classification parameters on the direct relationship between muscle strengthening physical activity volume and diastolic blood pressure.

VMSPA→ DBP	Sex	R-Sq	Effect	SE	*t*	*p*	LLCI	ULCI
All	Male	0.4975	−0.00043	0.00055	−0.78159	0.43534	−0.00152	0.00065
	Female		−0.00266	0.00135	−1.96323	0.05095	−0.00534	0.00001
Good CRF	Male	0.4444	−0.0004	0.0012	−0.3700	0.7132	−0.0027	0.0019
	Female		−0.0063	0.0357	0.1758	0.8613	−0.0657	0.0783
Low CRF	Male	0.5734	0.0006	0.0008	0.7746	0.4398	−0.0009	0.0021
	Female		−0.0028	0.0015	−1.8812	0.0619	−0.0058	0.0001

VMSPA, volume of muscle-strengthening physical activity; DBP, diastolic blood pressure; CRF, cardiorespiratory fitness; R-Sq, R-squared; SE, standard error; LLCI, lower limit 95% confidence interval; ULCI, upper limit 95% confidence interval.

Bold indicates statistical significant at *p*-value <0.05.

**Table 11:** Subgroup analysis with cardiorespiratory fitness classification parameters on the direct relationship between muscle strengthening physical activity volume and mean arterial pressure.

VMSPA → MAP	Sex	R-Sq	Effect	SE	*t*	*p*	LLCI	ULCI
All	Male	0.6236	0.00034	0.00053	0.65647	0.51224	−0.00070	0.00139
	Female		−0.00172	0.00130	−1.32430	0.18686	−0.00430	0.00084
Good CRF	Male	0.6286	−0.0003	0.0010	0.3261	0.7459	−0.0017	0.0023
	Female		−0.0035	0.0304	−0.1156	0.9085	−0.0647	0.0577
Low CRF	Male	0.6528	0.0014	0.0008	1.7527	0.0817	−0.0002	0.0029
	Female		−0.0015	0.0015	−0.9755	0.3309	−0.0045	0.0015

VMSPA, volume of muscle-strengthening physical activity; MAP, mean arterial pressure; CRF, cardiorespiratory fitness; R-Sq, R-squared; SE, standard error; LLCI, lower limit 95% confidence interval; ULCI, upper limit 95% confidence interval.

Bold indicates statistical significant at *p*-value <0.05.

According to the Tables 9–11, among 221 subjects, there were 58 subjects with good cardiorespiratory fitness (≥40 ml/kg/minute) and 163 with low cardiorespiratory fitness (<40 ml/kg/minute). In Table 9, it can be seen that for male subjects in the good fitness group, the relationship between the volume of muscle strengthening exercises and systolic blood pressure was not significant (*p*: 0.1057, 95% CI −0.0004 to 0.0040), compared to the group of all fitness levels (*p*: 0.00989, 95% CI 0.00046 to 0.00356) and in the low fitness group (*p*: 0.0124, 95% CI 0.0006 to 0.0052), which is significant. Comparing R2 among the three groups, we can see the good fitness group explains the variation in systolic blood pressure readings in the population by 75.44%, compared with 69.16% in the all fitness group and 65.39% in the low fitness group. As a result, in people with a high level of cardiorespiratory fitness, the relationship between the volume of muscle-strengthening exercises and blood pressure is not significant.

## 4. Discussion

### 4.1. Key result and interpretation

This study shows statistically that there is a significant positive relationship between MSPA and blood pressure, after entering gender and abnormal blood pressure as moderators. Different results appear when the two variables are not considered, where there is no significant direct effect between MSPA and BP. This shows that sex and abnormal pressure function as moderator variables in the relationship between MSPA and BP.

In previous studies that investigated the relationship between muscle strength and BP, there was a positive relationship between MS and BP. However, in the present study, the relationship between MS and BP was not significant. This could be influenced by many things, seeing as the number of samples and the age range used in previous studies were different. However, MSPA has a direct effect on SBP. In the components and domains of MSPA, it turns out that free weight also has a direct effect on SBP in men.

However, there was no indirect effect from the mediators studied. This shows that there are other mechanisms or factors other than physical fitness that can mediate the relationship between MSPA and blood pressure. We suspect that there are three things that can explain why there are differences in the effects of MSPA on BP in men and women.

First, hormone and autonomic response differences between gender. In women, there is hormone secretion, such as estrogen, which has an effect on nitric oxide synthesis, and provides a relaxing effect on peripheral resistance of blood vessels (30). In addition, it is well known that testosterone levels in women are lower than in men. Meanwhile, higher testosterone levels are associated with higher blood pressure (31).

It is well known that blood pressure is the product of cardiac output and peripheral resistance. Cardiac output is the product of the heart rate and stroke volume, where the heart rate component is strongly influenced by the work of the sympathetic and parasympathetic nerves. While, in resting conditions, young women have lower muscle sympathetic activity to support blood pressure than men (32, 33). This condition is different in older women, especially in menopausal conditions, where there is an accelerated process of sympathetic activity. This explains why, in resting conditions, women's heart rates tend to be lower than men's (34).

Women typically have a poorer capacity for arterial bed vasoconstriction than males do under specific circumstances following physical activity. Following exercise, women's vascular resistance is lower than men's. This could be the reason why women's blood pressure tends to drop more quickly and effectively after exercise than men's does (35).

Second, differences in metabolism during conditions of abnormal blood pressure. Because of the cross-sectional design of this study, it is not possible to determine whether the association between blood pressure and the amount of muscle-strengthening exercise is causative or allows for a two-way relationship. The increase in blood pressure may be due to adaptation to muscle-building activities, or the individual may already have high blood pressure before beginning resistance training. Regarding the relationship between muscle strengthening exercises and blood pressure, there is some evidence indicating that there is a disturbance of skeletal muscle oxygenation during exercise in adult subjects with hypertension (34). When compared to persons without hypertension, the adult population with hypertension has diminished functional sympatholysis. It has been demonstrated that hypertension people had reduced blood flow and oxygen delivery to functioning skeletal muscles. These effects were assessed in response to lower body negative pressure both at rest and during moderately intense handgrip exercises (34).

In addition, acute weight training will have an impact on the activation of muscle fibers due to alterations in skeletal muscle metabolism that occur in people with high blood pressure. In individuals with high blood pressure, there is a shift from oxidative metabolism to predominantly glycolytic metabolism under hypoxic conditions in skeletal muscle during weight training This increased use of energy from glycolysis will change the recruitment of type II muscle fibers to be dominant over type I muscle fibers (36, 37, 38, 39). It is clearly known that the size of type II muscle fibers is relatively larger than type I. This may underlie muscle strengthening exercises associated with increased strength and muscle mass, which can also be associated with high blood pressure.

Third, resistance training reduces arterial compliance and exacerbates aortic wave reflection, especially in young subjects (40, 41). In one study, interventions in young adults showed a 20% reduction in central arterial compliance after 2 months of resistance training. The occurrence of arterial stiffness certainly plays a role in increasing blood pressure. Stiffness can occur due to an intermittent increase in arterial blood pressure. This can change the structure of the arteries, especially components that play a role in load-bearing conditions, such as connective tissue collagen and elastin. Although resistant exercise has not been shown to change intima-media thickness, it does have the potential to change the quality of the arterial wall by causing fractures of the elastic lamellae. In addition, high-intensity resistance training is a powerful stimulus to the sympathetic nervous system. Chronic increases in the sympathetic nervous system can decrease arterial compliance through chronic restraint on the arterial wall via the greater sympathetic adrenergic vasoconstrictor tone (40).

Fourth, there are non-biological factors that can affect a person's health, including how a person's reasons for doing physical activity and how a person's body responds to physical activity and produces blood pressure output (42). One of them is social appearance anxiety. Social appearance anxiety is a feeling of distress related to other people's judgments of one's physical appearance. A young population that is physically active with the aim of losing weight significantly shows greater anxiety than someone who just wants to keep fit (43). In this study, an examination was carried out related to the mental well-being of the subjects, using the WEMWBS questionnaire. Mental well-being is a condition of being happy and satisfied, with low stress levels, physically and mentally healthy, and having a good quality of life. The WEMWBS can assess mental well-being but does not assess mental disorders. In fact, both mental well-being and mental disorders can affect blood pressure (36). Given the existence of psychological factors (such as anxiety and depression) in the relationship between physical activity and health outcomes, particularly in the young adult population, it is necessary to conduct research that focuses on identifying neurobehavioral factors that investigate one's goals and perspectives in doing physical activity and their relationship to health outcomes.

In addition to these four mechanisms, this study found that cardiorespiratory fitness level played a role in the relationship between the volume of muscle-strengthening exercises and blood pressure. It is known that there is a significant positive relationship between the volume of muscle strengthening exercises and systolic blood pressure in male subjects with high blood pressure. The volume of muscle strengthening exercises and systolic blood pressure relationship was not significant in male subjects with good fitness (p: 0.1057, 95% CI: −0.0004 to 0.0040). From these results, it can be concluded that a good level of cardiorespiratory fitness has a protective effect on blood pressure in the young adult population, especially in male subjects with high blood pressure. Therefore, someone at a young age who does muscle strengthening exercises is advised to maintain cardiorespiratory fitness by doing aerobics activities as a companion to muscle strengthening exercises.

### 4.2. Limitation

There are two limitations to this study. First, we know that a person's cardio-metabolic state is influenced by hereditary factors. Because this study focuses on functional outcomes, especially aspects of fitness, we do not carry out tests that lead to biomarkers or genetic characteristics. Second, because this research is not an interventional study, the conclusions in this study cannot prove a definite causal relationship between the independent variable and the outcome. For future research, lab-based RCTs can be conducted to learn more about the mechanisms, or research in the form of mediation analysis of behavioral-based cluster interventions can be conducted to see a more in-depth dose-response in the population.

## 5. Conclusion

This study investigates the relationship between MSPA and blood pressure in a young adult population, taking into account mediators, moderators, and various covariate variables. The results show that MSPA has a significant positive direct effect on systolic blood pressure, especially in the male population with high blood pressure and low cardiorespiratory fitness. It can be estimated that every 625 min of 4 MET MSPA per week is associated with an increase in systolic blood pressure of 5 mmHg in the male population that has high blood pressure. Seeing this, when exercising, the community, especially fitness practitioners, must consider the dose of exercise that is in accordance with a person's premorbid conditions. Looking at the results of the sensitivity analysis, the possibility of a harmful effect of MSPA on blood pressure can be minimized by increasing cardiopulmonary fitness by also doing aerobic activity in addition to MSPA. In addition, functional parameters such as physical fitness do not have a mediating effect on the relationship between MSPA and blood pressure, so it is necessary to study the mechanism to explain other pathways that can explain the pathway of the relationship between MSPA volume and blood pressure.

## Data Availability

The raw data supporting the conclusions of this article will be made available by the authors, without undue reservation.
